# Association of Maternal Nationality with Stillbirth and Infant Mortality: An Analysis of National Data in Japan

**DOI:** 10.31662/jmaj.2025-0544

**Published:** 2026-05-22

**Authors:** Tasuku Okui

**Affiliations:** 1 Medical Information Center, Kyushu University Hospital, Fukuoka City, Japan

**Keywords:** Japan, stillbirth, infant mortality, nationality, birth

## Abstract

**Introduction:**

This study investigated the association of maternal nationality with stillbirth and infant mortality in Japan.

**Methods:**

The Vital Statistics data in Japan from 2010 to 2023 were used, and singleton births born from 2010 to 2022 were used in the analysis of stillbirth and infant mortality. The maternal nationality consisted of Japan, Korea, China, the Philippines, Thailand, the United States (US), Brazil, Peru, and other countries in the analysis. Stillbirth and infant mortality rates were calculated by maternal nationalities. In addition, modified Poisson regression models were used to investigate the associations between the outcomes and maternal nationality, and residential area, parity, maternal age, marital status, and year of birth were adjusted.

**Results:**

Data on 12,287,030 live births and 33,968 stillbirths were analyzed. As a result, the stillbirth rate for American mothers was the highest, and that of “other countries” was the second highest. In addition, the infant mortality rate of American mothers was also the highest. The multivariate regression analysis revealed that mothers from the US and “other countries” had a significantly higher risk of stillbirth than Japanese mothers and mothers from the US had a significantly higher risk of infant mortality than Japanese mothers.

**Conclusions:**

It was suggested that support before and after childbirth is particularly necessary for American mothers in Japan.

## Introduction

Stillbirth and infant mortality are representative indicators of maternal and child health worldwide. The estimated stillbirth rate in Japan, calculated as the number of fetuses that died at ≥22 weeks of completed gestation per 1,000 births, decreased from 4.5 per 1,000 births in 1990 to 1.9 per 1,000 births in 2021 ^(1)^. The infant mortality rate, which also showed a decreasing trend over the decades, changed from 4.6 per 1,000 live births to 1.8 per 1,000 live births in 2023 ^(2)^. It is known that the risk of those outcomes varies depending on maternal factors such as parity, body mass index, and maternal age at the time of childbirth in Japan ^(3), (4), (5)^.

Maternal ethnicity and nativity are also known to be associated with stillbirth and infant mortality, and the associations were shown in some countries ^(6), (7), (8), (9), (10), (11), (12)^. In Japan, a study investigated the association of maternal nationality with fetal and infant mortality ^(13)^, demonstrating that fetal and infant mortality rates for Filipino mothers were the highest among Japanese, Korean, Chinese, Filipino, and Brazilian mothers. However, associations with maternal nationality such as Thailand and the United States (US), whose data were available in the Vital Statistics, were not investigated in the study. In addition, other maternal features such as age and marital status were not taken into account in the analysis. Moreover, data from 1995 to 2008 were used in the analysis, and it is meaningful to investigate the associations using more recent data. It is known that maternal nationality was associated with birth outcomes such as preterm birth, low birth weight, and macrosomia in the analysis of recent data in Japan ^(14), (15)^, and it is possible that there are some differences in stillbirth and infant mortality rates by maternal nationality. The number of births by people of non-Japanese nationalities has been increasing over the years in Japan ^(16)^, and it is meaningful to identify the nationalities of mothers who have higher risks of adverse birth outcomes.

In this study, we assessed the association of maternal nationality with stillbirth and infant mortality using national data in Japan.

## Materials and Methods

### Data source and data processing

Birth, fetal mortality, and mortality data from the Vital Statistics in Japan from 2010 to 2023 were used in the analysis. The data were provided by the National Statistics Center upon application on the basis of Article 33 of the Statistics Act in Japan. In addition, the data were provided to an on-site facility for the microdata of the official statistics in Japan, and we used an on-site facility to analyze the data. Specifically, a faculty member of a University could apply for the microdata, and we applied to the National Statistics Center for the microdata through e-Micro (https://www.e-stat.go.jp/microdata/, accessed on March 22, 2026). The microdata could be used in on-site facilities located in universities and administrative agencies upon permission of the National Statistics Center. The Vital Statistics data cover all the births, fetal deaths, and deaths which were notified to the local governments in Japan. In addition to data on age, date of birth, parity, nationality, and marital status of mothers, data on prefecture, municipality, sex, date of birth, birthweight, gestational age in weeks, and number of fetuses were used as birth data. For fetal mortality data, in addition to data on age, parity, nationality, and marital status of mothers, data on date of death, type of fetal death, gestational age in weeks, and number of fetuses were used. For mortality data, in addition to data on the date of birth of mothers, data on sex, date of death, prefecture, municipality, birthweight, gestational age in weeks, number of fetuses, and the indicator of infant mortality caused by diseases were used.

The definition of stillbirth varies depending on the studies, although it is common to define stillbirth as spontaneous fetal death occurring at ≥22 completed weeks of gestation in Japan ^(3), (17), (18)^. Therefore, that definition was also employed in this study. Infant mortality was defined as the mortality of infants within one year after birth. To attach the status of infant mortality for live birth data, data linkage was performed between live birth data and infant mortality data. The prefecture, municipality, infant’s sex, and dates of birth of infants and mothers were used as matching keys to merge the data. Only one-to-one matched cases were treated as infant mortality cases to minimize false matches. When multiple birth data were merged with one infant mortality data, birthweight and gestational weeks were also added to the matching keys in the linkage, and one-to-one matched cases considering birthweight and gestational weeks were also treated as infant mortality cases additionally. In addition, the second data linkage was conducted using live birth and infant mortality data that were unmatched in the first linkage because it is possible that residential address changed for some infants. Information on infant’s sex, dates of birth of infants and mothers, birthweight, and gestational weeks was used as matching keys in the second data linkage, and prefecture and municipality were not used because they might have changed before the infant’s death for some cases that were not matched in the first data linkage. Only cases that were matched one-to-one were treated as infant mortality in this matching. Because some of the characteristics of infants were not available for infant mortality due to external causes, we used data on disease-related infant mortality in this study.

Maternal nationality in the Vital Statistics data consists of Japan, Korea, China, the Philippines, Thailand, the US, the United Kingdom, Brazil, Peru, and other countries. The United Kingdom was included in the “other countries” in this study because the number of mothers from the United Kingdom and the number of stillbirths and infant mortality from those mothers were small. Maternal age was grouped into <20 years, 20-24 years, 25-29 years, 30-34 years, 35-39 years, and ≥40 years. According to parity, mothers were grouped into primiparous and multiparous women. Residential area was categorized into metropolitan areas, small and medium-sized urban areas, and non-urban areas. Metropolitan areas indicate cities designated by government ordinance and the special wards in Tokyo, and small and medium-sized urban areas indicate the other cities.

### Statistical analysis

Only singleton births were used in the analysis. Because infant mortality was used as an outcome, live births that occurred in 2010-2022 were used in the analysis of infant mortality. In addition, live births and stillbirths that occurred in 2010-2022 were used in the analysis of stillbirth in order to align the study population. Moreover, data without information for the first data linkage or those in which the residential area was outside Japan were omitted from the analysis.

We tallied the number of births by maternal nationality and maternal and infant characteristics. In addition, the stillbirth and infant mortality rates were calculated by maternal nationality. Moreover, regression models were used to investigate the associations between the outcomes and maternal nationality. A modified Poisson regression model was used in the analysis, and two types of models were applied for each outcome. The first model included only the maternal nationality and year of birth as explanatory variables to verify the crude associations. The second model included maternal nationality, residential area, parity, maternal age group, marital status, and the year of birth as explanatory variables. The risk ratio (RR), sandwich variance, 95% confidence intervals (CIs), and p-value for each outcome were calculated. Two-sided tests were conducted, and p < 0.05 was considered statistically significant. All the statistical analyses were conducted by R4.4 ^(19)^, and lmtest and sandwich were used as packages ^(20), (21)^. The statistics shown in this study were created by the author using data provided by the National Statistics Center, and those are not statistics published by the Ministry of Health, Labour and Welfare.

## Results

**Figure 1.**
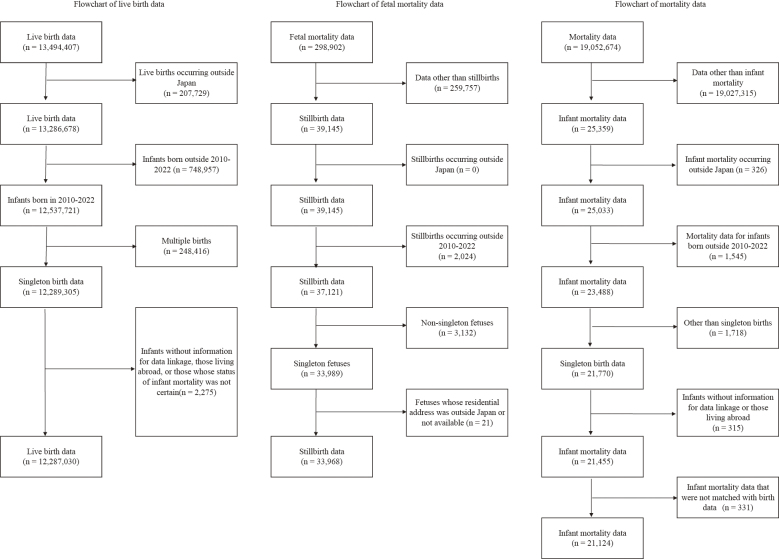
Flowchart of the selection process for birth, fetal mortality, and infant mortality data.

Figure 1 shows the flowchart of the selection process for birth, fetal mortality, and infant mortality data. Data on 12,287,030 live births, 33,968 stillbirths, and 21,124 infant deaths were used in the analysis. As a result of data linkage, most of the infant mortality data (21,124 among 21,455 infant deaths) were matched with live birth data.

Table 1 shows the number of births by maternal nationality and maternal and infant characteristics. The number of births varied largely by maternal nationality. The proportion of unmarried mothers varied largely by maternal nationality, and the proportions for Filipino, Brazilian, and Peruvian mothers were over 20% and particularly large. The preterm birth (<37 gestational weeks) rate for American mothers was 9.1% and was the highest. In addition, low birth weight (birthweight <2,500 g) rate for Filipino mothers was 8.9% and was the highest, and rate of macrosomia (birthweight ≥4,000 g) for American mothers was 7.3% and was the highest.

**Table 1.** Number (%) of Births by Maternal Nationality and Maternal and Infant Characteristics.

	Maternal nationality									
Characteristics	Japan	Korea	China	Philippines	Thailand*	United States	Brazil	Peru*	Other countries
Total	12,002,933 (100.0)	32,356 (100.0)	109,892 (100.0)	47,137 (100.0)	5,687 (100.0)	5,037 (100.0)	25,655 (100.0)	7,135 (100.0)	85,166 (100.0)
Residential area									
Metropolitan areas	3,654,497 (30.4)	17,867 (55.2)	56,713 (51.6)	12,622 (26.8)	1,789 (31.5)	1,880 (37.3)	3,126 (12.2)	1,066 (14.9)	34,727 (40.8)
Small and medium-sized urban areas	7,424,908 (61.9)	13,535 (41.8)	50,013 (45.5)	31,498 (66.8)	3,505 (61.6)	2,788 (55.4)	20,429 (79.6)	5,545 (77.7)	46,510 (54.6)
Non-urban areas	923,528 (7.7)	954 (2.9)	3,166 (2.9)	3,017 (6.4)	393 (6.9)	369 (7.3)	2,100 (8.2)	524 (7.3)	3,929 (4.6)
Maternal age group									
19 years or less	130,792 (1.1)	176 (0.5)	254 (0.2)	1,053 (2.2)	96 (1.7)	24 (0.5)	1,087 (4.2)	352 (4.9)	745 (0.9)
20-24 years	1,043,514 (8.7)	1,564 (4.8)	7,188 (6.5)	5,875 (12.5)	516 (9.1)	384 (7.6)	4,414 (17.2)	1,361 (19.1)	11,245 (13.2)
25-29 years	3,211,841 (26.8)	6,474 (20.0)	33,142 (30.2)	12,289 (26.1)	1,276 (22.4)	1,341 (26.6)	6,890 (26.9)	1,895 (26.6)	31,055 (36.5)
30-34 years	4,316,312 (36.0)	12,353 (38.2)	44,864 (40.8)	14,511 (30.8)	1,897 (33.4)	1,933 (38.4)	7,048 (27.5)	1,845 (25.9)	27,728 (32.6)
35-39 years	2,691,475 (22.4)	9,289 (28.7)	20,642 (18.8)	10,002 (21.2)	1,461 (25.7)	1,101 (21.9)	4,831 (18.8)	1,246 (17.5)	11,912 (14.0)
40 years or more	608,999 (5.1)	2,500 (7.7)	3,802 (3.5)	3,407 (7.2)	441 (7.8)	254 (5.0)	1,385 (5.4)	436 (6.1)	2,481 (2.9)
Marital status									
Married	11,723,562 (97.7)	31,135 (96.2)	107,185 (97.5)	36,894 (78.3)	5,120 (90.0)	4,879 (96.9)	18,916 (73.7)	5,025 (70.4)	81,180 (95.3)
Unmarried	279,371 (2.3)	1,221 (3.8)	2,707 (2.5)	10,243 (21.7)	567 (10.0)	158 (3.1)	6,739 (26.3)	2,110 (29.6)	3,986 (4.7)
Parity									
Primiparous	5,649,323 (47.1)	15,716 (48.6)	61,576 (56.0)	13,841 (29.4)	2,544 (44.7)	2,347 (46.6)	9,718 (37.9)	2,335 (32.7)	44,565 (52.3)
Multiparous	6,353,610 (52.9)	16,640 (51.4)	48,316 (44.0)	33,296 (70.6)	3,143 (55.3)	2,690 (53.4)	15,937 (62.1)	4,800 (67.3)	40,601 (47.7)
Gestational age									
36 weeks or less	588,663 (4.9)	1,519 (4.7)	4,598 (4.2)	3,813 (8.1)	396 (7.0)	458 (9.1)	1,937 (7.6)	-	4,950 (5.8)
37 weeks or more	11,411,332 (95.1)	30,821 (95.3)	105,271 (95.8)	43,276 (91.8)	5,274 (92.7)	4,566 (90.6)	23,650 (92.2)	-	80,129 (94.1)
Missing	2,938 (0.0)	16 (0.0)	23 (0.0)	48 (0.1)	17 (0.3)	13 (0.3)	68 (0.3)	-	87 (0.1)
Birth weight									
<2,500 g	1,011,696 (8.4)	2,034 (6.3)	4,622 (4.2)	4,186 (8.9)	-	411 (8.2)	1,869 (7.3)	-	5,903 (6.9)
2,500-3,999 g	10,893,301 (90.8)	29,790 (92.1)	101,605 (92.5)	42,136 (89.4)	-	4,243 (84.2)	23,009 (89.7)	-	76,887 (90.3)
4,000 g or more	96,123 (0.8)	517 (1.6)	3,649 (3.3)	794 (1.7)	-	367 (7.3)	712 (2.8)	-	2,316 (2.7)
Missing	1,813 (0.0)	15 (0.0)	16 (0.0)	21 (0.0)	-	16 (0.3)	65 (0.3)	-	60 (0.1)

*The values of gestational age and birth weight were not shown because there were categories whose values were very small and were not allowed to be disclosed.

Table 2 shows the number of live births, stillbirths, and infant deaths and the rates of stillbirth and infant mortality by maternal characteristics. The stillbirth and infant mortality rates for unmarried mothers were 8.0 per 1,000 births and 4.3 per 1,000 live births, respectively, and those for married mothers were 2.6 per 1,000 births and 1.7 per 1,000 live births, respectively. The stillbirth rate of Japanese mothers was 2.7 per 1,000 births. In contrast, the stillbirth rate for mothers from the US was 14.3 per 1,000 births and was the highest, and the rate for “other countries” was 4.5 per 1,000 births and was the second highest. In addition, the infant mortality rate for Japanese mothers was 1.7 per 1,000 live births, while that for mothers from the US was 7.3 per 1,000 live births.

**Table 2.** Number of Live Births, Stillbirths, and Infant Deaths and the Rates of Stillbirth and Infant Mortality by Maternal Nationality.

	Live births	Stillbirth		Infant mortality	
Characteristics	Number	Number	Number per 1,000 births	Number	Number per 1,000 live births
Residential area				
Metropolitan areas	3,774,282	10,005	2.6	6,182	1.6
Small and medium-sized urban areas	7,577,403	21,328	2.8	13,238	1.7
Non-urban areas	935,345	2,635	2.8	1,704	1.8
Maternal age group				
19 years or less	134,033	546	4.1	512	3.8
20-24 years	1,073,140	2,921	2.7	2,227	2.1
25-29 years	3,298,369	7,834	2.4	4,528	1.4
30-34 years	4,417,358	11,133	2.5	6,459	1.5
35-39 years	2,743,268	8,691	3.2	5,367	2.0
40 years or more	620,862	2,843	4.6	2,031	3.3
Marital status				
Married	11,982,390	31,506	2.6	19,821	1.7
Unmarried	304,640	2,462	8.0	1,303	4.3
Parity				
Primiparous	5,784,859	17,106	2.9	9,077	1.6
Multiparous	6,502,171	16,862	2.6	12,047	1.9
Nationality				
Japan	11,970,131	32,802	2.7	20,501	1.7
Korea	32,265	91	2.8	56	1.7
China	109,621	271	2.5	168	1.5
Philippines	46,938	199	4.2	118	2.5
Thailand	5,668	19	3.3	12	2.1
United States	4,965	72	14.3	36	7.3
Brazil	25,548	107	4.2	60	2.3
Peru	7,114	21	2.9	15	2.1
Other countries	84,780	386	4.5	158	1.9

Table 3 shows the results of the regression analyses investigating the associations of maternal nationality with stillbirth and infant mortality. In the model adjusting for the year of birth only, mothers from the Philippines, the US, Brazil, and “other countries” had a significantly higher risk of stillbirth than Japanese mothers. In contrast, in the model adjusting also for other factors, only mothers from the US and “other countries” had a significantly higher risk of stillbirth than Japanese mothers, and the adjusted RRs were 5.20 (95% CI: 4.14-6.55) and 1.70 (95% CI: 1.54-1.88), respectively.

**Table 3.** Results of the Regression Analyses Investigating the Associations of Maternal Nationality with Stillbirth and Infant Mortality.

	Model 1*		Model 2†	
Outcome/nationality	Adjusted RR (95% CI)	p-value	Adjusted RR (95% CI)	p-value
Stillbirth				
Japan	Reference		Reference	
Korea	1.02 (0.83-1.26)	0.822	0.97 (0.79-1.19)	0.767
China	0.90 (0.80-1.02)	0.098	0.93 (0.82-1.04)	0.204
Philippines	1.54 (1.34-1.77)	<0.001	1.14 (0.99-1.32)	0.062
Thailand	1.22 (0.78-1.92)	0.379	1.04 (0.67-1.63)	0.857
United States	5.25 (4.17-6.61)	<0.001	5.20 (4.14-6.55)	<0.001
Brazil	1.53 (1.26-1.85)	<0.001	1.07 (0.88-1.29)	0.501
Peru	1.08 (0.70-1.65)	0.740	0.73 (0.48-1.12)	0.150
Other countries	1.68 (1.52-1.86)	<0.001	1.70 (1.54-1.88)	<0.001
Infant mortality				
Japan	Reference		Reference	
Korea	1.00 (0.77-1.30)	0.997	0.97 (0.75-1.26)	0.823
China	0.90 (0.77-1.05)	0.170	0.97 (0.83-1.12)	0.660
Philippines	1.47 (1.22-1.76)	<0.001	1.09 (0.91-1.31)	0.349
Thailand	1.24 (0.70-2.18)	0.458	1.07 (0.61-1.88)	0.821
United States	4.28 (3.09-5.93)	<0.001	4.30 (3.10-5.95)	<0.001
Brazil	1.38 (1.07-1.77)	0.013	0.98 (0.76-1.27)	0.889
Peru	1.23 (0.74-2.03)	0.430	0.83 (0.50-1.39)	0.485
Other countries	1.13 (0.97-1.32)	0.123	1.16 (0.99-1.35)	0.067

CI: confidence interval; RR: risk ratio. *Year of birth was adjusted. †Residential area, parity, maternal age group, maternal marital status, and year of birth were adjusted.

In the model adjusting for the year of birth only, mothers from the Philippines, the US, and Brazil had a significantly higher risk of infant mortality than Japanese mothers. In contrast, in the model adjusting also for other factors, only mothers from the US had a significantly higher risk of infant mortality than Japanese mothers, and the adjusted RR was 4.30 (95% CI: 3.10-5.95).

## Discussion

This study demonstrated statistically significant associations of some maternal nationalities with stillbirth and infant mortality in Japan, and the results changed when adjusting for some characteristics such as maternal marital status. Herein, we discuss the possible reasons for these association, their implications, and limitations of the study.

The risk of stillbirth and infant mortality was higher among Filipino and Brazilian mothers, compared to Japanese mothers in the analyses that did not adjust for residential area, the mother’s marital status, maternal age, and parity. In contrast, no statistically significant associations were shown in the analyses that adjusted for those characteristics. The results of this study showed that proportions of unmarried mothers were particularly higher among Filipino and Brazilian mothers than among Japanese mothers. The maternal unmarried status was shown to be associated with infant mortality and spontaneous fetal mortality in Japan ^(5), (22)^, and our study also showed that unmarried mothers had a higher stillbirth and infant mortality rates. Therefore, it is possible that higher proportion of unmarried mothers has led to an increase in the rates of the outcomes of interest among Filipino and Brazilian mothers.

No maternal nationality had a significantly lower risks of stillbirths and infant mortality than Japanese mothers, while some mothers from foreign nationalities had higher risks than Japanese mothers. A systematic review and meta-analysis indicated that immigrant women had higher risks of stillbirths and neonatal mortality than native-origin women ^(23)^, and the result of our study was similar to it. Specifically, American mothers had higher risks of stillbirth and infant mortality than Japanese mothers regardless of the analysis methods employed, and Americans had the highest risks. The stillbirth rate in the US was estimated to be high among countries with high socioeconomic status and was higher than that in Japan ^(1)^. In addition, the infant mortality rate in the US was 5.44 per 1,000 live births in 2021, which was also higher than that in Japan ^(24)^, and the rates for Asian and Black mothers were the lowest and highest, respectively, among the maternal racial groups. Moreover, it is known that the infant mortality and stillbirth rates of immigrant mothers were lower than those of native mothers in the US ^(9), (25)^. Our study also showed that preterm birth rate for American mothers was the highest. Preterm birth rate among singleton births in the US was 8.7% in 2023 and was similar to that of our study ^(26)^. A study conducted in the US showed preterm birth rate was lower among non-US-born mothers compared with US-born mothers ^(27)^, while there were heterogeneities in the results depending on race and ethnicity. The rate of macrosomia was also high among American mothers in our study. The rate among singleton births in the US was approximately 8.0% and was relatively similar to our study ^(28)^. Preterm birth was shown to be a major risk factor of infant mortality in Japan ^(5)^, and macrosomia or large-for-gestational-age status was also shown to be related to stillbirths ^(29), (30)^. Therefore, there is a possibility that American nationality was associated with stillbirths and infant mortality through those adverse birth outcomes.

Mothers from “other countries” also had a higher risk of stillbirth than Japanese ones. Although the reason is uncertain, the relatively low stillbirth rate in Japan in the world might be related to the significantly higher risk of “other countries”. The number of births in which maternal nationality was “other countries” was increasing over the years in Japan ^(16), (31)^. The specific nationalities of “other countries” were not investigated in the vital statistics survey, and it is meaningful to scrutinize them and also their birth outcomes in future studies

According to the World Bank Open data, the infant mortality rates per 1,000 live births in Korea, China, the Philippines, Thailand, the US, Brazil, and Peru from 2010 to 2023 were approximately 3, 5-13, 20-25, 8-12, 6, 13-16, and 14-17, respectively ^(32)^. In addition, according to the Global Burden of Disease study, stillbirth rates per 1,000 births at 22 gestational weeks or later for those countries in 2015 were 2.6, 9.2, 9.8, 5, 5.1, 10.5, and 12.7, respectively ^(1)^. Therefore, the stillbirth and infant mortality rates for each maternal nationality in Japan tended to be lower than those in their native countries, except for the US. When calculating the rates in this study, multiple births and infant mortality caused by external causes were not considered, which could account for the difference. In contrast, the difference in rates tended to be higher in developing countries, possibly due to the difference in the quality of medical care between Japan and the native country.

According to our findings, American mothers had the highest risk of stillbirth and infant mortality in Japan, implying that these mothers should be supported before and after childbirth. In contrast, it is not certain whether American nationality is the determinant of birth outcomes because there are other possible confounding factors, including barriers to healthcare access, differences in healthcare utilization, and body mass index. It would also be meaningful to probe into the characteristics of American mothers, such as physical features, utilization of antenatal care, and race in future studies. In addition, it was shown that the proportion of unmarried persons was high among Filipino and Brazilian mothers, which is one possible reason for the higher risk of stillbirth in those mothers. It is important to investigate the reasons for the high proportion of unmarried persons in mothers of those nationalities.

As a limitation of the study, we could not obtain data on the length of the stay in Japan or data on race, ethnicity, and nativity for mothers. Healthy migrant effects existed for mortality among immigrants in Japan ^(33)^, and those factors can affect the outcomes. In addition, we did not analyze infant mortality by external causes because it was difficult to exactly identify those births from all the birth data. In contrast, the number of infant mortality by external causes was small compared with that of infant mortality by diseases. Specifically, infant mortality by external causes accounted for 6.3% of all cases of infant mortality in 2010-2023 ^(31)^. Therefore, our result is not generalizable to infant mortality by external causes, while infant mortality by diseases accounted for a large part of infant mortality in Japan. However, it will be meaningful to survey the rate of infant mortality by external causes by maternal nationality in the future. Moreover, some of the infant mortality cases were not matched with live birth data during data linkage. Furthermore, the findings in our study apply only to singleton births, and differences in multiple birth rates depending on maternal nationalities could influence the overall infant mortality rate. In contrast, we used the nationwide Vital Statistics data in the analysis, and the results represent the overall associations between the outcomes and maternal nationality in Japan.

In conclusion, it was shown that stillbirth and infant mortality rates for American mothers were the highest among the major maternal nationalities represented in Japan. The multivariate regression analysis also showed that mothers from the US and “other countries” had a significantly higher risk of stillbirth than Japanese mothers and that mothers from the US had a significantly higher risk of infant mortality than Japanese mothers. The results suggested the greater need for support to American mothers in Japan before and after childbirth. In addition, it was also suggested that it is meaningful to scrutinize maternal nationalities of the “other countries” in future studies.

## Article Information

### Acknowledgments

Enago has proofread the manuscript.

### Author Contributions

Conceptualization: Tasuku Okui. Data curation: Tasuku Okui. Formal analysis: Tasuku Okui. Methodology: Tasuku Okui. Funding acquisition: None. Writing- original draft: Tasuku Okui. Writing - review & editing: Tasuku Okui.

### Conflicts of Interest

None

### Approval by Institutional Review Board

This study was conducted in accordance with the Declaration of Helsinki and was approved by the Kyushu University Institutional Review Board for Clinical Research (No. 25059). Additionally, informed consent was not required for this study because we used the official statistics data that were provided from the National Statistics Center in Japan on the basis of the Statistics Act.
